# Probiotic *Lactobacillus johnsonii* Reduces Intestinal Inflammation and Rebalances Splenic Treg/Th17 Responses in Dextran Sulfate Sodium-Induced Colitis

**DOI:** 10.3390/antiox14040433

**Published:** 2025-04-03

**Authors:** Hao-Yu Liu, Shicheng Li, Kennedy Jerry Ogamune, Peng Yuan, Xinyu Shi, Wael Ennab, Abdelkareem A. Ahmed, In Ho Kim, Ping Hu, Demin Cai

**Affiliations:** 1 College of Animal Science and Technology, Yangzhou University, Yangzhou 225009, China; haoyu.liu@yzu.edu.cn (H.-Y.L.); mx120220855@stu.yzu.edu.cn (S.L.); mh24022@stu.yzu.edu.cn (K.J.O.); mz120231586@stu.yzu.edu.cn (P.Y.); mz120241643@stu.yzu.edu.cn (X.S.); 008568@yzu.edu.cn (W.E.); pinghu@yzu.edu.cn (P.H.); 2 Joint International Research Laboratory of Agricultural & Agri-Product Safety, The Ministry of Education of China, Yangzhou University, Yangzhou 225009, China; 3 Department of Veterinary Biomedical Sciences, Botswana University of Agriculture and Natural Resources, Gaborone P.O. Box 100, Botswana; aabdallah@buan.ac.bw; 4 Department of Animal Resource and Science, Dankook University, Cheonan 31116, Republic of Korea; inhokim@dankook.ac.kr

**Keywords:** inflammatory bowel disease, *Lactobacillus johnsonii*, gut microbiota, spleen, Treg/Th17

## Abstract

Inflammatory bowel disease (IBD), a chronic inflammatory disorder of the gastrointestinal tract, is frequently complicated by extraintestinal manifestations such as functional hyposplenism. Increasing evidence highlights its pathogenesis as a multifactorial interplay of gut dysbiosis, intestinal barrier dysfunction, and dysregulated immune responses. While probiotics, particularly *Lactobacillus* spp., have emerged as potential therapeutics for IBD, restoring intestinal homeostasis, their systemic immunomodulatory effects remain underexplored. Here, we investigated the protective role of *Lactobacillus johnsonii* N5 in DSS-induced colitis, focusing on inflammation inhibition and splenic T cell regulation. Pretreatment with *L. johnsonii* N5 significantly attenuated colitis severity, as evidenced by preserved body weight, reduced disease activity index, and prevention of colon shortening. N5 suppressed colonic pro-inflammatory factors such as TNF-α, Il-1b, Il-6, and CXCL1, while elevating anti-inflammatory IL-10 at both mRNA and protein levels. Transcriptomic analysis of the spleen revealed that N5 mediated the downregulation of inflammatory pathways, including the IL-17 and TNF signaling pathways, as well as the HIF-1 signaling pathway, and modulated the metabolic pathway of oxidative phosphorylation. Flow cytometry analysis demonstrated that N5 rebalanced splenic Treg/Th17 responses by expanding the Treg population and reducing the production of IL-17A in Th17 cells. Notably, Th17-associated IL-17A positively correlated with intestinal pro-inflammatory mediators, emphasizing the role of Th17 cells in driving colitis. In contrast, splenic Treg abundance positively correlated with colonic IL-10 levels, suggesting a link between systemic immune regulation and intestinal anti-inflammatory responses. Our study underscores the therapeutic potential of targeting gut–immune crosstalk through probiotics, thereby offering valuable insights for developing live bacterial-based interventions for IBD and other inflammatory disorders.

## 1. Introduction

Inflammatory bowel disease (IBD), encompassing Crohn’s disease (CD) and ulcerative colitis (UC), is a chronic relapsing inflammatory disorder of the gastrointestinal (GI) tract. Beyond its intestinal pathology, IBD is frequently associated with extraintestinal manifestations, including functional hyposplenism—a complication reported in 9–54% of patients [1,2]. With its global incidence rising, particularly in newly industrialized regions, IBD has become a significant cause of morbidity and imposes substantial healthcare burdens [3]. Current therapeutic strategies, including anti-inflammatory agents, immunosuppressants, and biologics, often yield suboptimal outcomes, underscoring the urgent need for alternative strategies [4]. The etiology of IBD is multifactorial, but central to its pathogenesis is the disruption of intestinal homeostasis—a delicate equilibrium maintained by mucosal barrier integrity, immune tolerance, and symbiotic interactions between the host and gut microbiota [3]. Dysbiosis, traditionally characterized by reduced microbial diversity, depletion of beneficial taxa (e.g., Firmicutes), and expansion of pathobionts (e.g., Proteobacteria), disrupts this equilibrium, driving inflammation through aberrant immune activation and epithelial barrier dysfunction [5,6]. These processes amplify the production of pro-inflammatory cytokines such as TNF-α and IL-1β, while impairing regulatory pathways, perpetuating intestinal injury and ultimately leading to a “leaky gut” in IBD [4,5,7].

Indeed, the gut microbiota plays a pivotal role in modulating host immunity and barrier function [8]. Animal models and clinical studies have demonstrated that germ-free conditions attenuate colitis, whereas microbiota transplantation from IBD patients can transfer disease susceptibility, highlighting the causative role of microbiota in IBD [9,10]. Recent insights into intestinal microecology suggest that dysbiosis in IBD reflects not only compositional shifts but also a state of weakened host control over microbial communities, enabling inflammation [7,11]. In IBD, epithelial barrier injury further intensifies this vicious cycle: disrupted tight junction proteins (e.g., ZO-1, occludin) permit bacterial translocation, activating innate and adaptive immune cells [12,13] and amplifying inflammatory cascades [14]. Consequently, therapeutic strategies targeting microbial ecology, barrier restoration, and immune modulation have emerged as promising approaches to disrupt this self-reinforcing pathology [15].

Probiotics, defined as live microorganisms that confer health benefits to the host, have emerged as a promising treatment option for IBD management [16]. Among these, *Lactobacillus*—a highly diverse genus of bacteria—has been extensively studied for its anti-inflammatory and immunomodulatory properties, with specific strains demonstrating efficacy in alleviating colitis in preclinical mouse models and in IBD patients [17,18]. For instance, *Lactobacillus reuteri* mitigates colitis in murine models by fortifying mucosal barriers and suppressing pro-inflammatory cytokines [6,19,20,21]. Similarly, *Lactobacillus rhamnosus* GG alleviates diarrhea and inflammation through microbiota modulation and immune regulation [22]. Our previous study identified *Lactobacillus johnsonii* N5, a strain isolated from stress-resistant animals [23], and *L. reuteri* [24], both of which prevent dextran sulfate sodium (DSS)-induced colitis by reducing inflammation and exhibiting T cell-mediated probiotic effects in Peyer’s patches (PPs). Mechanistically, *Lactobacillus* and its metabolites promote dendritic cell (DC)-mediated IL-10 production and regulatory T (Treg) cell differentiation, suggesting a role in immune tolerance [14,23]. However, the systemic immunomodulatory effects of probiotics, particularly their influence on extraintestinal lymphoid organs such as the spleen, remain underexplored.

The spleen is a key hub for microbial control, orchestrating systemic immune regulation of bloodborne antigens through T cell responses. Transient bacterial translocation from the gut typically resolves harmlessly when splenic function is intact [25]. Tregs play a critical role in this process: their local and systemic effects—including active suppression, clonal deletion, and anergy of effector T cells—prevent hypersensitivity to intestinal antigens, thereby maintaining immune tolerance [26,27]. Oral tolerance, which can be induced by repeated low-dose antigen exposure (a regimen analogous to probiotic administration), relies heavily on Treg-mediated suppression [27]. However, functional hyposplenism, a common complication of IBD and other gastrointestinal disorders, compromises these protective mechanisms, increasing infection risk and potentially exacerbating immune dysregulation [1]. In support of this, splenectomy in mice induces intestinal barrier defects and gut dysbiosis, while DSS-treated splenectomized mice develop more severe colitis and bacteremia [25], indicating a possible spleen–gut correlation. In colitis, imbalances in splenic T cell populations—specifically, heightened Th17 responses (pro-inflammatory) [28] and diminished Treg activity (anti-inflammatory)—correlate with disease severity [29]. T helper 17 (Th17) cells, driven by IL-6 and IL-23, secrete IL-17A, exacerbating intestinal inflammation, while Tregs suppress effector T cells via IL-10 and TGF-β [30]. Restoring the Treg/Th17 balance represents a therapeutic avenue, yet the role of probiotics in modulating this axis remains unclear.

Therefore, in this study, we utilized the DSS-induced murine colitis model—a robust and reproducible system for studying intestinal inflammation [24,31], and investigated the protective mechanisms of *L. johnsonii* N5 against colitis, focusing on intestinal cytokine regulation and splenic T cell responses. We consolidated the probiotic potential of *L. johnsonii* N5, revealing its anti-inflammatory effects in the colon. Furthermore, we demonstrated its systemic immunomodulatory capacity against DSS-induced Treg/Th17 imbalance using transcriptomic analysis and flow cytometry of the spleen. By linking gut microbiota interventions to extraintestinal immune regulation, this study highlights the potential of probiotics like *L. johnsonii* N5 as multi-target therapies for IBD.

## 2. Materials and Methods

### 2.1. Animals

**Figure 1:**
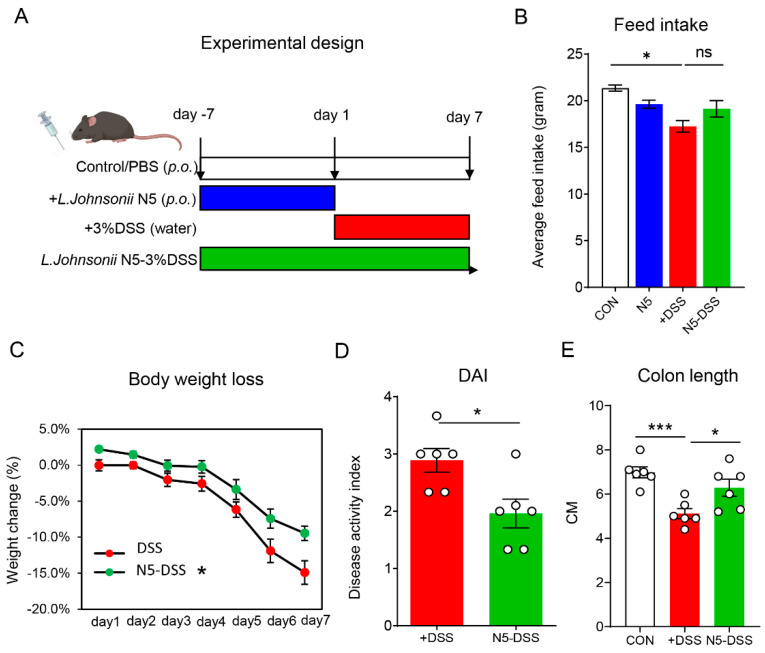
*Lactobacillus johnsonii* N5 protects against dextran sulphate sodium (DSS)-induced colitis in mice. (**A**) Schematic diagram of the experimental design: colitis was induced by providing wild-type C57BL/6 mice with 3% DSS in drinking water for 7 days; *Lactobacillus johnsonii* N5 (10^9^ CFU/mL) was given daily for 7 days (N5 group) or 14 days starting 7 days prior to DSS treatment (N5-DSS group). (**B**) Average feed intake for different groups (gram). (**C**) Changes in body weight (%) for DSS and N5-DSS groups. (**D**) Disease activity index (DAI) for DSS and N5-DSS. (**E**) Colon length (cm) was measured in CON, DSS and N5-DSS groups. Data are displayed as mean ± SEM (n = 6 mice per group). ns, not significant, * p < 0.05, *** p < 0.001 using two-tailed Student’s *t* test or ANOVA with Tukey’s post hoc test.

All animal experiments in this study were conducted in strict accordance with the ethical guidelines established by the Animal Care and Use Committee of Yangzhou University [SYXK (SU) 2021-0026]. Male C57BL/6 wild-type mice, aged 8 weeks, were obtained from the Jiangsu Laboratory Animals Science Center. The mice were housed under specific pathogen-free conditions, with controlled ambient parameters: temperature set at 22 °C, humidity at 50%, and a 12 h light/dark cycle. They were provided unrestricted access to water and a standard laboratory chow diet. The experimental design is shown in Figure 1A. Mice with comparable body weights were randomly assigned to four experimental groups (n = 6): the control group (CON), the group pretreated with *Lactobacillus johnsonii* N5 (N5), the group treated with dextran sulfate sodium (DSS), and the group receiving both N5 and DSS (N5-DSS). Colitis was induced by administering drinking water containing 3% (wt/vol) DSS (MW 36,000–50,000, Yeasen, Shanghai, China) for 7 days. Disease activity was monitored daily following previously established protocols [6]. The evaluation included assessments of body weight changes, stool consistency (classified as normal, loose, or diarrhea), and fecal occult blood tests to detect rectal bleeding. For the N5 and N5-DSS groups, *L. johnsonii* N5 was freshly prepared and administered orally at a dosage of 10^8^ CFU (10^9^ CFU/mL) per mouse per day. In the N5 group, treatment lasted for 7 consecutive days, while in the N5-DSS group, treatment began 7 days prior to DSS administration and continued for a total of 14 days.

### 2.2. Sample Collection

At the end of the experiment, all mice were humanely euthanized via intraperitoneal injection of 200 μL of 0.9% sodium pentobarbital solution. Distal colonic tissues and spleen samples were immediately processed, frozen in liquid nitrogen, and stored at −80 °C for further analysis. Colon length was measured, and the spleens were freshly processed into single-cell suspensions for flow cytometry analysis.

### 2.3. Bacteria Preparation

The *Lactobacillus johnsonii* N5 strain, sourced from our laboratory and deposited at the China Center for Type Culture Collection (CCTCC, NO: M2023104), was cultivated in MRS broth (Oxoid) at 37 °C for 20 h as previously described [23]. Bacteria were harvested during the early stationary phase, washed with PBS, concentrated 100-fold, and preserved at −80 °C in a cryoprotectant solution (containing 0.82 g K_2_HPO_4_, 0.18 g KH_2_PO_4_, 0.59 g sodium citrate, 0.25 g MgSO_4_·7 H_2_O, and 172 mL of 87% glycerol, diluted to a final volume of 1000 mL with deionized water).

### 2.4. Enzyme-Linked Immunosorbent Assay (ELISA)

The concentrations of cytokines, including interleukin (IL)-10, tumor necrosis factor-α (TNF-α), and chemokine (C-X-C motif) ligand 1 (CXCL1) in the distal colon, were measured using ELISA kits (Beyotime, Shanghai, China) following manufacturer’s protocols.

### 2.5. Quantitative Real-Time PCR

Total RNA was extracted from distal colonic and spleen tissues using Trizol reagent (Invitrogen, 15596026, Waltham, MA, USA) following the manufacturer’s instructions. RNA quantification was performed using a NanoDrop 1000 spectrophotometer (F-3100, Suizhen, Hangzhou, China). Subsequently, cDNA synthesis was conducted using HiScript II Q RT SuperMix (Vazyme Biotech, R222-01, Nanjing, China) with 1 μg of RNA. Quantitative reverse transcription polymerase chain reaction (qRT-PCR) was performed using AceQ qPCR SYBR Green Master Mix (Vazyme Biotech, Q111-02, Nanjing, China) on an ABI QuantStudio 3 Real-Time PCR system. The expression level of *Gapdh* was used as an internal reference, and relative gene expression levels were calculated using the 2^−ΔΔCT^ method. Primer sequences for qPCR are listed in Table 1.

### 2.6. Transcriptomic Analysis

Total RNA was isolated from the spleen. The RNA concentration was determined using a NanoDrop 2000 spectrophotometer (Thermo Fisher Scientific, San Francisco, CA, USA), while the RNA quality was assessed with the Agilent Bioanalyzer 2100 system (Agilent Technologies, Santa Clara, CA, USA). Subsequently, RNA-seq libraries were prepared using the Illumina TruSeq RNA Sample Prep Kit (Illumina, San Diego, CA, USA). High-throughput sequencing libraries were generated and sequenced on the Illumina HiSeq 2000 platform (BGI Tech, Wuhan, China). The raw sequencing data were processed using SOAPnuke (v1.5.6) to generate clean reads, which were eventually stored in FASTQ format. High-quality clean reads were aligned to the GRCm39 reference genome using Bowtie2 (v2.5.0). For further analysis, cufflinks software version 2.2.1 was employed to calculate fragments per kilobase of exon per million mapped fragments (FPKM) values. Differential gene expression analysis was performed using DESeq2 version 1.32.0, with differentially expressed genes identified based on a threshold of log_2_ (fold change) > 1 and adjusted *p*-value < 0.05.

### 2.7. Flow Cytometry Analysis

Single-cell suspensions were prepared by mechanically dissociating spleen tissue. Red blood cells were lysed using 0.2% NaCl for 30 s and neutralized with 1.6% NaCl. Samples were then washed, filtered through a 40 μm cell strainer, and resuspended in PBS containing 0.05% fetal bovine serum (FBS) and 2 mM EDTA. Cells were blocked with anti-CD16/32 antibody (2.4G2, BD Bioscience, Franklin Lakes, NJ, USA) and stained for T cell subsets using anti-CD3 (17A2) and anti-CD4 (GK1.5) antibodies (BioLegend, San Diego, CA, USA). Dead cells were excluded using the Zombie Aqua™ Fixable Viability Kit (BioLegend, CA, USA). Intracellular markers, including forkhead box P3 (FoxP3; MF-14), RORγt (AFKJS-9), and IL-17a (TC11-18H10.1), were detected after fixation and permeabilization using the True-Nuclear™ Transcription Factor Buffer Set (BioLegend, CA, USA). Data were acquired on a CyAn ADP7 flow cytometer (Beckman Coulter, Brea, CA, USA) and analyzed using FlowJo version 10.0.8 (Tree Star, Inc., San Francisco, CA, USA).

### 2.8. Data Availability

RNA-seq data are available in the SRA under BioProject accession number PRJNA1175388.

### 2.9. Statistical Analysis

Statistical analysis was carried out using GraphPad Prism version 9 (GraphPad Software, Inc., San Diego, CA, USA). Two-tailed Student’s *t*-test or one-way analysis of variance (ANOVA) with Tukey’s post hoc test were used for normally distributed continuous variables. The Gene Set Enrichment Analysis (GSEA) was performed using GSEA 4.1.0 to identify significantly enriched pathways. The differences in pathway abundance were annotated by referring to the Kyoto Encyclopedia of Genes and Genomes (KEGG) database. The analysis was conducted using STAMP software v2.1.3, and statistical significance was determined using Welch’s *t*-test, with the Benjamini–Hochberg correction applied to adjust values for multiple comparisons. Volcano plot and Venn diagram analysis were performed using the OmicShare tools, a free online platform for data analysis (https://www.omicshare.com/tools (accessed on 19 September 2024)). Data are displayed as mean ± SEM and significant differences were evaluated by Tukey’s multiple comparison tests; *p* < 0.05 was considered significant.

## 3. Results

### 3.1. Lactobacillus johnsonii N5 Protects Against DSS-Induced Colitis in Mice

To evaluate whether oral administration of *Lactobacillus johnsonii* N5 exerts beneficial effects on the host, 8-week-old male C57BL/6 mice were used to establish a colitis model by administering 3% dextran sulfate sodium (DSS) in drinking water for 7 days (Figure 1A). Compared to the control group, DSS-treated mice exhibited a significant reduction in average feed intake (Figure 1B, *p* < 0.05). However, pretreatment with *L. johnsonii* N5 did not prevent this reduction in feed intake (*p* > 0.05). Notably, mice that received *L. johnsonii* N5 pretreatment demonstrated strong protective effects, preserving body weight and reducing the disease activity index (DAI) in response to DSS-induced colitis (Figure 1C,D, *p <* 0.05). Furthermore, N5 pretreatment effectively mitigated DSS-induced colon shortening, (Figure 1E, *p* < 0.05), a hallmark of colonic inflammation.

### 3.2. Lactobacillus johnsonii N5 Modulates Colonic Cytokine and Chemokine Levels in DSS-Induced Colitis in Mice

**Figure 2:**
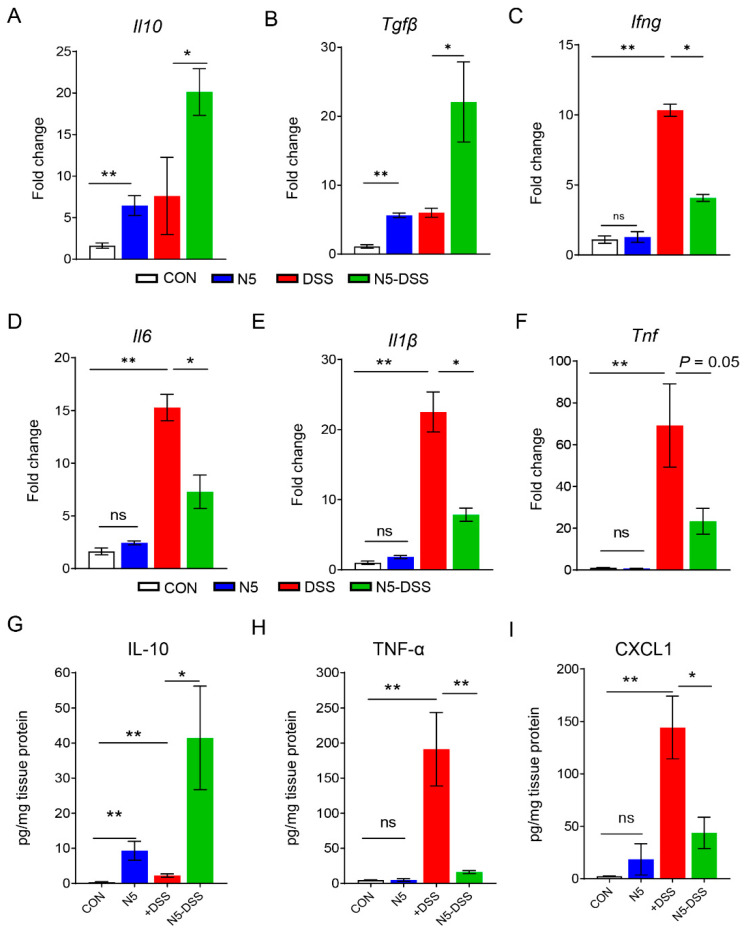
*Lactobacillus johnsonii* N5 modulates the distal colonic cytokine/chemokine levels against dextran sulfate sodium-induced colitis in mice. (**A**–**F**) qRT-qPCR analysis of anti-inflammatory cytokines (*Il10* and *Tgfβ*) and pro-inflammatory cytokines (*Ifng*, *Il6*, *Il1β* and *Tnf*) gene expression in the distal colon mucosa. The data were normalized to the expression of *GAPDH.* (**G**–**I**) Concentrations of colonic IL-10, TNF-α and CXCL1 levels (pg/mg tissue protein). Data are displayed as mean ± SEM, (n = 6 per group). ns, not significant, * *p* < 0.05, ** *p* < 0.01 using ANOVA with Tukey’s post hoc test.

Quantitative RT-qPCR analysis of the distal colonic mucosa revealed that pretreatment with *Lactobacillus johnsonii* N5 significantly upregulated the gene expression of anti-inflammatory cytokines, including *Il10* (*p* < 0.05) and *Tgfβ* (*p* < 0.05), compared to the DSS-treated group (Figure 2A,B). Conversely, DSS treatment markedly increased the gene expression of pro-inflammatory cytokines, including *Ifng* (*p* < 0.01), *Il6* (*p* < 0.01), *Il1β* (*p* < 0.01), and *Tnf* (*p* < 0.01), compared to the control group (Figure 2C–F). Notably, N5 pretreatment significantly reduced the expression levels of *Ifng* (*p* < 0.05), *Il6* (*p* < 0.05), and *Il1β* (*p* < 0.05) in DSS-treated mice.

Further analysis of protein levels demonstrated that IL-10 was significantly elevated in the distal colon of the N5-DSS group compared to the DSS group (Figure 2G, *p* < 0.05). In contrast, the levels of TNF-α (Figure 2H, *p* < 0.01) and CXCL1 (Figure 2I, *p* < 0.01) were significantly higher in DSS-treated mice than in healthy controls. Importantly, N5 pretreatment restored the levels of distal colonic TNF-α (*p* < 0.01) and CXCL1 (*p* < 0.05) in DSS-treated mice to near-normal levels. Finally, in the N5-only group, both mRNA and protein levels of IL-10 were significantly increased (*p* < 0.01), while no significant effects were observed on the expression of pro-inflammatory cytokines or CXCL1 compared to the control group (*p* > 0.05).

### 3.3. Lactobacillus johnsonii N5 Regulates the Splenic Transcriptome Against DSS-Induced Colitis in Mice

**Figure 3:**
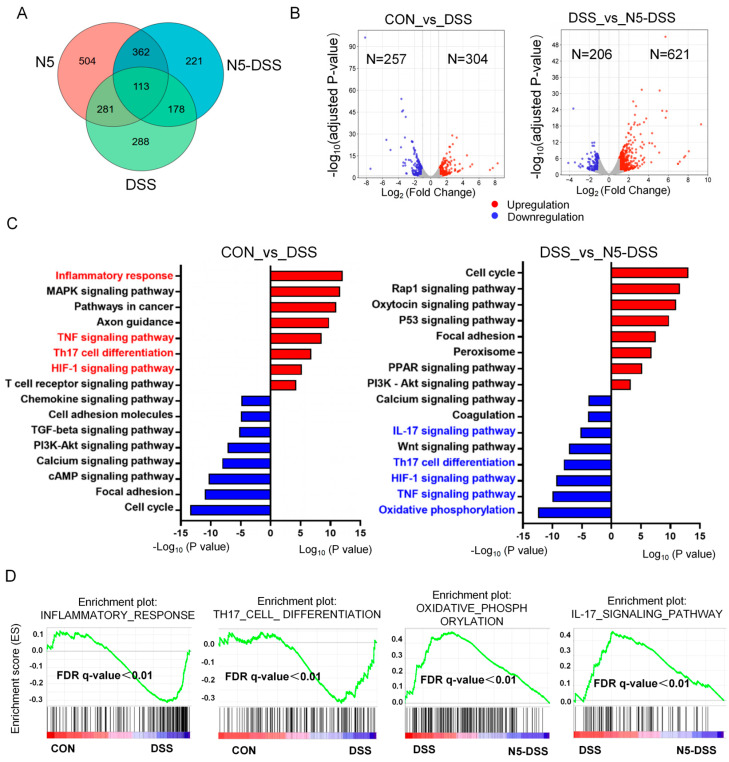
*Lactobacillus johnsonii* N5 regulates the splenic transcriptome against dextran sulfate sodium-induced colitis in mice. (**A**) Venn diagram of genes differentially expressed (Log_2_ expression, fold change > 1) in the spleen of mice for DSS vs. CON, N5 vs. CON, and N5-DSS vs. DSS, determined by RNA-seq analysis. (**B**) Volcano plot visualization of the differentially expressed genes for DSS vs. CON, and N5 + DSS vs. DSS. (**C**) The enrichment pathways analyzed by the KEGG for DSS vs. CON and DSS vs. N5-DSS. (**D**) GSEA plots depicting the enrichment of genes altered in Inflammatory response pathway, Th17 cell differentiation pathway, oxidative phosphorylation pathway and IL-17 signaling pathway (FDR, false discovery rate). Hypergeometric test, with the Benjamini–Hochberg *p*-value correction (FDR, false discovery rate), was applied.

To investigate whether, and if so, how *L. johnsonii* N5 exerts regulatory effects on extraintestinal organs during DSS-induced colitis, we performed a comprehensive RNA-Seq analysis on spleen samples. The spleen was chosen as a key extraintestinal immune organ because it plays a central role in systemic immune responses and serves as a critical hub for immune cell activation and cytokine production during inflammatory conditions [32]. The Venn diagram illustrates the differentially expressed genes (|Log_2_ (fold change) | > 1) between the DSS and control groups, the N5 and control groups, and the N5-DSS and DSS groups (Figure 3A). Volcano plot visualization revealed 304 upregulated and 257 downregulated genes when comparing the DSS group to the control group, and 621 upregulated and 206 downregulated genes when comparing the N5-DSS group to the DSS group (Figure 3B). These findings indicate a substantial extraintestinal effect of N5 on the splenic transcriptome in response to DSS-induced colitis.

Functional annotation of the differentially expressed transcripts is shown in Figure 3C. Kyoto Encyclopedia of Genes and Genomes (KEGG) pathway analysis revealed that the DSS treatment enriched pathways associated with inflammation, oxidative stress and immunity, including the ‘inflammatory response’, ‘TNF signaling pathway’, ‘Th17 cell differentiation’ and ‘HIF-1 signaling pathway’, among others. In contrast, pretreatment with N5 downregulated pathways such as the ‘IL-17 signaling pathway’, ‘Th17 cell differentiation’, ‘TNF signaling pathway’, ‘HIF-1 signaling pathway’ and ‘oxidative phosphorylation’ compared to the DSS group. In agreement with the KEGG analysis, Gene Set Enrichment Analysis (GSEA) further demonstrated that the DSS group upregulated pathways related to the inflammatory response and Th17 cell differentiation compared to the control group. Conversely, the N5-DSS group exhibited downregulation of pathways such as oxidative phosphorylation and the IL-17 signaling pathway (Figure 3D).

**Figure 4:**
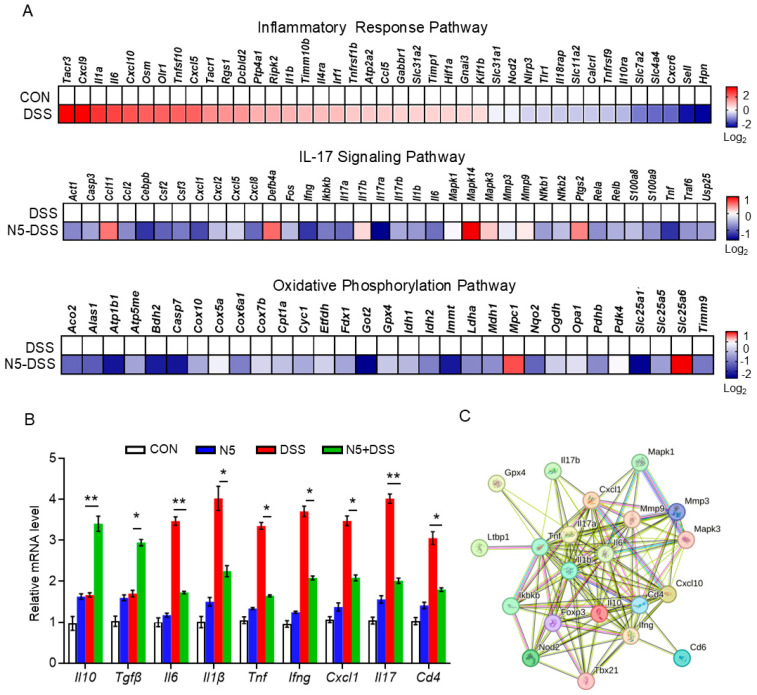
*Lactobacillus johnsonii* N5 regulates the expression of genes involved in the inflammatory response and the IL-17 signaling pathway in the spleen against dextran sulfate sodium-induced colitis in mice. (**A**) Heatmaps depicting the enrichment of genes upregulated or downregulated in the inflammatory response pathway, IL-17 signaling pathway and oxidative phosphorylation pathway. (**B**) qRT-qPCR quantification of anti-inflammatory cytokine (*Il10* and *Tgfβ*) and pro-inflammatory cytokine (*Ifng*, *Il6*, *Il1β*, *Tnf*, *Hif1a*, *Il17* and *Cd4*) gene expression in the spleen. (**C**) The interactions among the inflammatory response and IL-17 signaling pathway key proteins involved in the transcriptional regulation were predicted by STRING-ELIXIR analysis. Data are displayed as mean ± SEM (n = 6 mice per group). * p < 0.05, ** p < 0.01 using ANOVA with Tukey’s post hoc test.

Further transcriptomic heatmap analysis revealed that DSS treatment significantly upregulated the gene expressions of inflammatory response pathways, including *Cxcl9*, *Il6*, *Cxcl10*, *Il1b*, *Hif1a*, *Rgs1*, *Osm* and *Irf1* (Figure 4A, upper panel). Conversely, N5 pretreatment significantly downregulated the gene expressions of the IL-17 signaling pathway, including *Act1*, *Casp3*, *Fos*, *Ifng*, *Ikbkb*, *Il17ra* and *Tnf* (Figure 4A, middle panel) and oxidative phosphorylation pathway, compared to the DSS group, including *Acos2*, *Atp1b1*, *Bgh2*, *Casp7*, *Got2* and *Immt* (Figure 4A, lower panel), respectively. The expression patterns identified in the transcriptomic analysis of anti-inflammatory (*Il10* and *Tgfβ*) and pro-inflammatory genes were quantitatively assessed using qRT-PCR (Figure 4B). Consistent with the transcriptomic findings, N5 pretreatment significantly restored the expression levels of *Il6*, *Il1β*, *Tnf*, *Ifng*, *Hif1a*, *Il17* and *Cd4* against DSS treatment (*p* < 0.05). In addition, STRING-ELIXIR analysis demonstrated interactions among key factors of the inflammatory pathway and the IL-17 signaling pathway, including *Il17a*, *Tnf*, *Il6*, and *Il10* (Figure 4C).

### 3.4. Lactobacillus johnsonii N5 Modulates the Spleen Treg/Th17 Responses Against DSS-Induced Colitis

**Figure 5:**
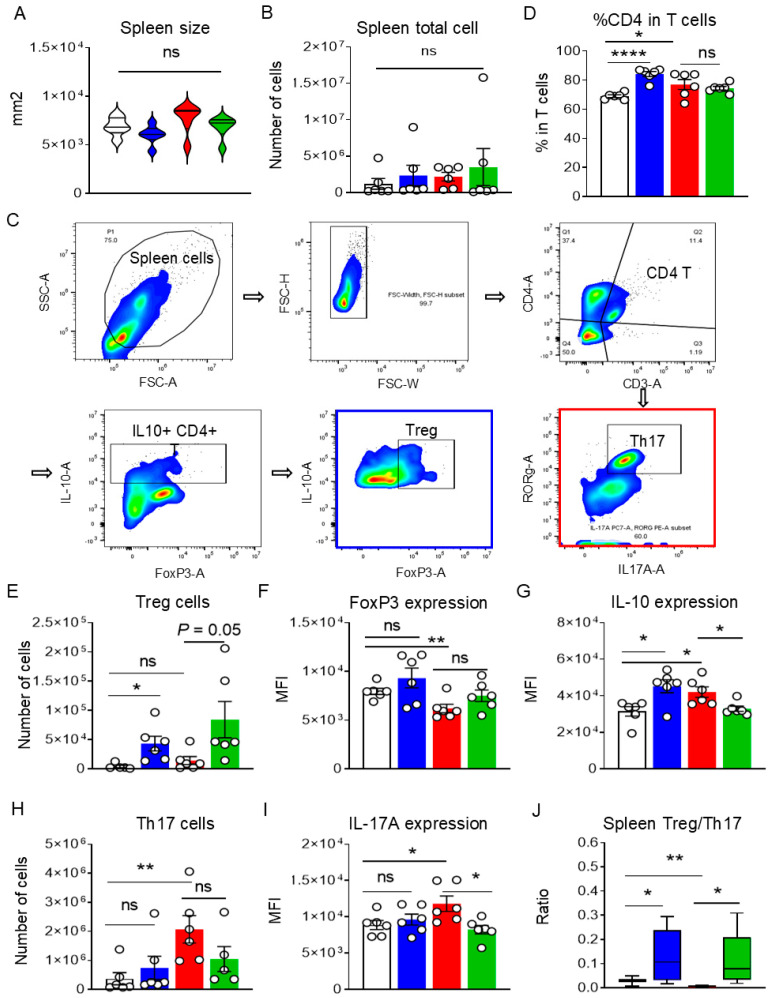
*Lactobacillus johnsonii* N5 modulates the spleen Treg/Th17 responses against dextran sulfate sodium-induced colitis. The size of spleen (mm^2^) (**A**) and total number of cells per spleen in mice (**B**) were recorded. (**C**) The staining and gating strategy of cell subsets isolated from spleen. (The single-cell suspension was prepared, separated and stained with different surface markers.) Upon flow cytometry analysis, doublets were removed and the live CD4^+^ T cells were further studied. (**D**) The percentage of CD4^+^ T cells in the spleen. (**E**,**F**) The number of Th17 cells and their IL-17A expression (MFI) of spleen in mice. (**G**–**I**) The number of regulatory T (Treg) cells, their FoxP3 expression (MFI) and IL-10 expression (MFI) in the spleen. (**J**) The ratio of Treg/Th17 in the spleen. Data are displayed as mean ± SEM (n = 6 mice per group). ns, not significant, * *p* < 0.05, ** *p* < 0.01, **** *p* < 0.001 using ANOVA with Tukey’s post hoc test.

Based on the observed changes in the splenic transcriptome in response to *L. johnsonii* N5 and/or DSS, we investigated the effects of N5 on Th17 vs. Treg responses in the spleen during DSS-induced colitis using flow cytometry. First, no significant differences were observed between the DSS and N5-DSS groups in terms of spleen size (Figure 5A, *p* > 0.05) or total cell count (Figure 5B, *p* > 0.05). However, compared to the control group, the N5 treatment significantly increased the percentage of CD4_+_ T cells within the total T-cell population (Figure 5D, *p* < 0.0001), as did DSS treatment (*p* < 0.05). Notably, no significant difference was observed between these two groups in the percentage of CD4_+_ T cells (*p* > 0.05).

Further analysis revealed that DSS treatment specifically increased the absolute number of Th17 cells in the spleen compared to healthy controls (Figure 5E, *p* < 0.01), along with elevated expression of the effector cytokine IL-17A (Figure 5F, *p* < 0.05). In contrast, N5 pretreatment did not alter the population of Th17 cells (*p* > 0.05) but significantly reduced IL-17A expression in the DSS-treated mice (*p* < 0.05). Meanwhile, N5 treatment modulated Treg populations in the spleen (Figure 5G–I). Specifically, N5 significantly increased the absolute number of Tregs compared to both the control and DSS-treated groups (*p* ≤ 0.05), and enhanced their expression of IL-10 (*p* < 0.05). Conversely, DSS treatment reduced the expression of the Treg transcription factor FoxP3 compared to the control group (*p* < 0.01), an effect that was not reversed by N5 pretreatment (*p* > 0.05). Finally, the DSS group exhibited a significantly lower Treg/Th17 ratio compared to the control group (Figure 5J, *p* < 0.01). In contrast, N5-associated treatments resulted in a significantly higher Treg/Th17 ratios compared to both the control (*p* < 0.05) and DSS groups (*p* < 0.05).

**Figure 6:**
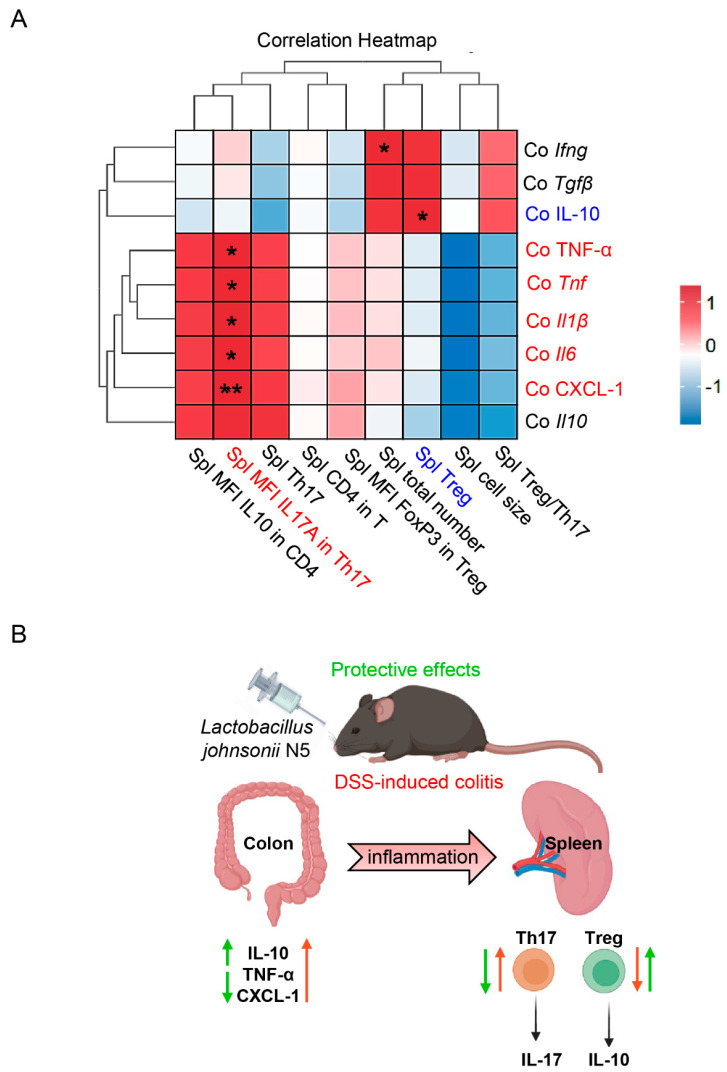
Correlation heatmap and schematic illustration demonstrating that *Lactobacillus johnsonii* N5 can modulate colonic inflammation and the splenic Treg/Th17 balance response. (**A**) Heatmap of Spearman’s correlation between changes in the splenic T cell populations and the inflammatory cytokine levels in the distal colon. Color ranges from blue (negative correlation) to red (positive correlation). (**B**) Schematic illustration of the protective effects of *Lactobacillus johnsonii* N5 against dextran sulfate sodium-induced colitis in mice. * *p* < 0.05, ** *p* < 0.01 using in the correlation heatmap.

To further explore the relationship between systemic immune responses and intestinal inflammation, Spearman correlation analysis was performed. Strong correlations were identified between changes in the colon and spleen (Figure 6A): IL-17A expression in splenic Th17 cells positively correlated with colonic levels of TNF-α and CXCL-1 (*p* < 0.05), as well as with the gene expression of pro-inflammatory cytokines such as *Tnf*, *Il6*, and *Il1β* (*p* < 0.05). Additionally, colonic *Il1β* expression was positively associated with total cell numbers in the spleen (*p* < 0.05). In contrast, the number of splenic Treg cells showed a positive correlation with colonic IL-10 production (*p* < 0.05). Together, these findings suggest that the protective effects of *Lactobacillus johnsonii* N5 on intestinal inflammation and spleen immune responses are interconnected (Figure 6B).

## 4. Discussion

Inflammatory bowel disease presents a complex challenge in gastrointestinal health, necessitating multifactorial therapeutic approaches [33]. Probiotics, particularly *Lactobacillus* species, have emerged as promising candidates due to their ability to modulate gut microbiota, enhance intestinal barrier integrity, and regulate immune responses, effectively alleviating IBD symptoms in preclinical models and clinical settings, including UC patients [18,19]. In this study we demonstrate that *Lactobacillus johnsonii* N5, a strain isolated from stress-resistant animals [34], confers robust protective effects against DSS-induced colitis by reducing intestinal inflammation and rebalancing systemic T cell responses, highlighting its potential as a multi-target therapeutic agent.

Central to IBD pathogenesis lies the disruption of intestinal homeostasis, where dysregulated cytokine networks drive tissue damage [4]. Our findings align with prior studies indicating that *Lactobacillus* species mitigate colitis by suppressing pro-inflammatory mediators [6,19,21]. Specifically, pretreatment with *L. johnsonii* N5 significantly reduced distal colonic expression of TNF-α, IL-1β, IL-6, and CXCL1, while elevating anti-inflammatory IL-10 levels. This cytokine shift correlated with preserved colon length and reduced disease severity, suggesting that N5 reinforces mucosal tolerance by dampening inflammatory cascades. Notably, N5’s ability to upregulate IL-10, even in the absence of DSS challenge, underscores its inherent immunomodulatory properties, priming the gut for resilience. During this process, DCs may play a pivotal role in bridging luminal antigen exposure to T cell responses, balancing pro-inflammatory and tolerogenic immunity [26]. Accordingly, *Lactobacillus* strains, such as *L. reuteri* [35], *L. johnsonii* and *L. rhamnosus* GG enhance IL-10 production via DC activation [14,36,37], promoting Treg differentiation and suppressing pathogenic Th17 responses. Our previous work also demonstrated that peroral administration of *L. reuteri* reduces pro-inflammatory CD11b^+^CD11c^+^ DCs in mesenteric lymph nodes (MLNs), key sources of inflammatory cytokines—while expanding Foxp3^+^CD4^+^ T cells during colitis [21]. Similarly, *L. johnsonii* N5 pretreatment stimulates MHCII^+^ DCs in PPs for antigen presentation and expands the tolerogenic CD103^+^ DC subset, ultimately increasing the Treg population in healthy mice [23]. Mechanistically, a recent study identified a specialized RORγt^+^ DC subset in MLNs that expresses CCR7, αv integrin (a TGF-β activator), enabling these cells to relay commensal-derived signals and direct Treg differentiation [38]. Although our study did not assess splenic DC changes in response to N5, their contribution to the observed splenic Treg expansion is plausible.

Importantly, our study demonstrates that *L. johnsonii* N5 exhibits significant systemic immunomodulatory properties, particularly in the spleen—a key hub for systemic immunity [32]. Transcriptomic analysis revealed that N5 downregulated splenic inflammatory pathways, including IL-17 and TNF signaling, while modulating HIF-1 signaling and oxidative phosphorylation against DSS challenge. This might point to a link between oxidative stress and mitochondrial dysfunction, which is known to drive Th17 cell reprogramming and amplify inflammatory responses [39,40]. In our study, DSS-induced colitis skewed splenic T cells and was characterized by elevated Th17 cell frequencies and increased IL-17A production. In contrast, N5 pretreatment reversed these changes, leading to the downregulation of pro-inflammatory genes such as *Cxcl9, Il6,* and *Hif1a*, while restoring metabolic homeostasis. These findings align with emerging evidence linking immune cell metabolism to inflammatory outcomes [32,39]. The suppression of IL-17 signaling is particularly noteworthy, as Th17-driven inflammation is a hallmark of IBD [30]. In adult and pediatric patients of IBD, a pathological transformation from Tregs to Th17 in blood is reported, indicating an impaired systemic immunosuppressive function [18,41]. Interestingly, our flow cytometry analysis further demonstrated that N5 rebalanced splenic T cell responses by expanding Tregs and reducing IL-17A production in Th17 cells. This shift in the Treg/Th17 ratio—a key determinant of immune tolerance—correlated with diminished colonic inflammation [19,42,43,44], highlighting a gut–spleen axis through which probiotics exert systemic immunomodulation. The positive association between splenic Treg abundance and colonic IL-10 levels further supports the notion that N5 enhances anti-inflammatory feedback loops spanning intestinal and systemic compartments.

The mechanisms underlying N5’s systemic effects may involve gut-derived signals transmitted via migratory immune cells from draining lymph nodes or through microbial metabolites [18,19,38,45,46]. *Lactobacillus* species produce metabolites such as lactic acid, indole-3-lactic acid, and short-chain fatty acids (SCFAs; e.g., acetate, propionate, butyrate), as well as bile salt hydrolases, enzymes that convert secondary bile acids [18,19,45]. These metabolites can circulate to extraintestinal sites, mediating systemic effects. For instance, *Lactobacillus-*derived phenylacetic acid upregulates intestinal PPAR-γ signaling, ameliorating metabolic dysfunction and adiposity in a microbiota-dependent manner [47]. SCFAs, in particular, directly promote Treg differentiation by acting as histone deacetylase (HDAC) inhibitors, enhancing IL-10 production [48,49], or migrating to bone marrow to induce tolerogenic antigen presenting cells (APCs), thereby reducing effector T cell recruitment and tissue inflammation [45], while *Lactobacillus*-associated bile acids like ursodeoxycholic acid (UDCA), isoDCA, and isoLCA modulate the Treg/Th17 balance in the ileum and liver, as demonstrated in nonalcoholic steatohepatitis (NASH) models [50]. Although our study did not directly profile metabolites, the observed splenic transcriptomic changes—notably in oxidative phosphorylation pathways—align with metabolic reprogramming of T cells, a key mechanism in immune regulation [39,40]. Furthermore, we have previously demonstrated that N5-induced expansion of CD103^+^ DCs in PPs may prime Treg differentiation in gut-associated lymphoid tissues, enabling their migration to systemic sites like the spleen [23].

Our findings align with and extend prior research on *Lactobacillus*-mediated immune regulation. At the molecular level, *Lactobacillus rhamnosus* reduces the colonic Th17/Treg ratio in DSS-induced colitis through TLR2-dependent JAK-STAT signaling [18,51], while *L. casei* M2S01 ameliorates colitis by restoring gut microbiota homeostasis, enhancing Treg activation and boosting IL-10 production via suppression of the canonical NF-κB pathway [52]. Beyond the gut, *Lactobacillus* species exert systemic immunomodulatory effects, on distant organs, such as the lungs. In Clec7a^–/–^ mice, peroral administration of *L. murinus* promotes Treg recruitment and immunosuppressive function not only in the intestine but also in the bronchoalveolar lavage fluid during ovalbumin-induced allergic airway inflammation, which is dependent on dectin-1 signaling [53]. Additionally, in a high-fat-diet-induced NASH model, *Lactobacillus*-associated secondary bile acids regulate hepatic Treg/Th17 differentiation and IL-17/PPAR signaling pathways, conferring liver protection [50]. Building on these insights, our study advances the field by linking *L. johnsonii* N5’s local anti-inflammatory effects to splenic immune reprogramming, providing a holistic view of its therapeutic potential. The observed correlation between splenic Treg/Th17 ratios and colonic cytokine levels underscores the gut–systemic immune axis, suggesting that probiotics targeting this axis could ameliorate both intestinal and extraintestinal complications of IBD.

While our findings highlight the therapeutic potential of *L. johnsonii* N5 in DSS-induced colitis, several limitations warrant consideration. First, this study relies exclusively on the DSS murine model, which exhibits inherent variability in inflammatory outcomes due to differences in host microbiota composition [31], underscoring the challenges of translating murine findings to human colitis, where microbiota–host interactions are far more heterogeneous. Second, while we focused on cytokine and T cell profiling, our study did not assess clinically relevant biomarkers of human IBD (e.g., fecal calprotectin, serum CRP, or IL-6), limiting direct translational relevance. Human IBD involves complex interactions between host genetics, adaptive immunity, and environmental factors that murine models only partially recapitulate. Finally, metabolomic profiling of intestinal lumen contents and systemic circulation is needed to elucidate how N5-derived metabolites mediate their anti-inflammatory and Treg-promoting effects.

In conclusion, *L. johnsonii* N5 represents a promising probiotic strategy for IBD management, capable of suppressing intestinal inflammation and rebalancing systemic T cell responses. By bridging gut microbiota interventions to extraintestinal immune regulation, this study highlights the potential of probiotics as multi-target therapeutic agents. Future research should prioritize translational validation in human IBD cohorts, incorporating multi-omics profiling (e.g., metabolomics, single-cell transcriptomics) to unravel N5-derived metabolite–immune crosstalk. Such efforts could pave the way for microbiome-based precision therapies, offering safer alternatives to conventional immunosuppressive regimens.

## Figures and Tables

**Table 1:** Real-time PCR primer sequences used in the current study.

Name	Primer Sequences (5′-3′)	Accession Number
*Il10*	F: CGGGAAGACAATAACTGCACC R: CGGTTAGCAGTATGTTGTCCA	NM_010548.2
*Il6*	F: AGCCCACCGGGAACGA R: GGACCGAAGGCGCTTGT	NM_031168.2
*Il1β*	F: CCACAGACCTTCCAGGAGAATG R: GTGCAGTTCAGTGATCGTACAGG	NM_008361.4
*Il17*	F: TCAACCCGATTGTCCACCAT R: GAGTTTAGTCCGAAATGAGGCTG	NM_010552.3
*Tnfα*	F: ATGAGCACTGAAAGCATGATCC R: GAGGGCTGATTAGAGAGAGGTC	NM_013693.3
*Ifng*	F: CAGCAACAGCAAGGCGAAAAAG R: TTTCCGCTTCCTGAGGCTGGA	NM_008337.4
*Hif1a*	F: CTATGGAGGCCAGAAGAGGGTAT R: CCCACATCAGGTGGCTCATAA	NM_010431.3
*Cd4*	F: TCTGGAACTGCACCGTGAC R: CCGTGATAGCTGTGCTCTGA	NM_013488.3
*Tgfβ*	F: GGCCAGATCCTGTCCAAGC R: GTGGGTTTCCACCATTAGCAC	NM_011577.2
*Gapdh*	F: GTCTCCTCTGACTTCAACAGCG R: ACCACCCTGTTGCTGTAGCCA	NM_008084.4

## Data Availability

RNA-seq data are available in the SRA under BioProject accession number PRJNA1175388.
